# CD4 T cell epitope specificity determines follicular versus non-follicular helper differentiation in the polyclonal response to influenza infection or vaccination

**DOI:** 10.1038/srep28287

**Published:** 2016-06-22

**Authors:** Zackery A. G. Knowlden, Andrea J. Sant

**Affiliations:** 1 David H. Smith Center for Vaccine Biology and Immunology, Department of Microbiology and Immunology, University of Rochester Medical Center, Rochester, NY, USA

## Abstract

Follicular helper T cells (Tfh) are essential for B cell production of high-affinity, class-switched antibodies. Much interest in Tfh development focuses on the priming environment of CD4 T cells. Here we explored the role that peptide specificity plays in the partitioning of the polyclonal CD4 T cell repertoire between Tfh and NonTfh lineages during the response to influenza. Surprisingly, we found that CD4 T cells specific for different epitopes exhibited distinct tendencies to segregate into Tfh or NonTfh. To alter the microenvironment and abundance, viral antigens were introduced as purified recombinant proteins in adjuvant as native proteins. Also, the most prototypical epitopes were expressed in a completely foreign protein. In many cases, the epitope-specific response patterns of Tfh vs. NonTfh persisted. The functional TcR avidity of only a subset of epitope-specific cells correlated with the tendency to drive a Tfh response. Thus, we conclude that in a polyclonal CD4 T cell repertoire, features of TcR-peptide:MHC class II complex have a strong deterministic influence on the ability of CD4 T cells to become a Tfh or a NonTfh. Our data is most consistent with at least 2 checkpoints of Tfh selection that include both TcR affinity and B cell presentation.

Follicular helper T cells (Tfh) represent an essential link between two arms of the adaptive immune system – CD4 T cell and B cell responses. This specialized differentiation state of CD4 T cells is necessary for the initiation and maintenance of the germinal center reaction that results in high-affinity, class-switched immunoglobulin production by plasma cells that have undergone affinity maturation and establishment of B cell memory^1^,^2^,^3^,^4^. Previous studies examining the factors contributing to the differentiation of a naïve CD4 T cell into Tfh have primarily focused on the role of cytokines, chemokines and the local microenvironment^5^,^6^,^7^, with early studies focusing heavily on the polarizing effects of IL-6 (mice), IL-12 (humans) and IL-21^8^,^9^,^10^. Coordination of signaling early in differentiation, especially signals through the ICOS-ICOSL pathway, has been shown to lead to upregulation of the Tfh-associated transcription factor Bcl6 as well as a chemokine receptor essential for entry into the B cell follicle, CXCR5^11^, with a concomitant decrease in CCR7 expression^6^,^12^. IL-2 signaling through CD25 has been demonstrated to have an antagonistic effect on Tfh factors, causing an increase in Blimp-1 expression as well as Tbet, both of which preclude a transition to the Tfh phenotype, while cementing a role as “NonTfh” effector cells^11^,^13^,^14^,^15^,^16^.

The role of T cell receptor signaling in commitment to this lineage has been less explored. Tfh are a unique T cell population, in that there is a requirement for sequential interactions with distinct populations of antigen presenting cells (APC), both dendritic cells (DC) and B cells^17^. The final commitment to the Tfh lineage is heavily dependent on interaction with B cells in the follicle^11^,^18^,^19^, through the provision of essential costimulation (ICOS and SLAM)^11^,^19^,^20^,^21^.

The role of TCR-peptide:MHC interactions in dictating commitment to the Tfh lineage has been the subject of several studies^22^,^23^,^24^, and have generally supported the view that high affinity and/or optimal dwell time may promote the selection of the Tfh pathway of differentiation. However, antigen specificity, and the relationship with and effects it has upon differentiation into follicular helpers or non-follicular helper (NonTfh) effector cells has not been examined in the context of a polyclonal CD4 T cell response in a complex antigenic environment such as an active infection. Herein, we describe our efforts to understand how the endogenous T cell repertoire responds to multiple independent epitopes during influenza infection and how the antigen specificity of the response influences the distribution of CD4 T cell follicular helpers or non-follicular helper effector cells. We show that selection into the Tfh pathway is dictated by the T cell specificity for the peptide epitope itself. In contexts ranging from the complex milieu of influenza infection, to vaccination with purified recombinant influenza proteins or heterologous protein constructs, in many cases, the intrinsic relationship of the pMHC:TCR complex is sufficient to confer effector outcome (Tfh vs. NonTfh) upon the polyclonal repertoire.

## Results

### Tfh and NonTfh cells in mice exhibit prototypical phenotypic markers and kinetics post influenza infection

**Figure 1:**
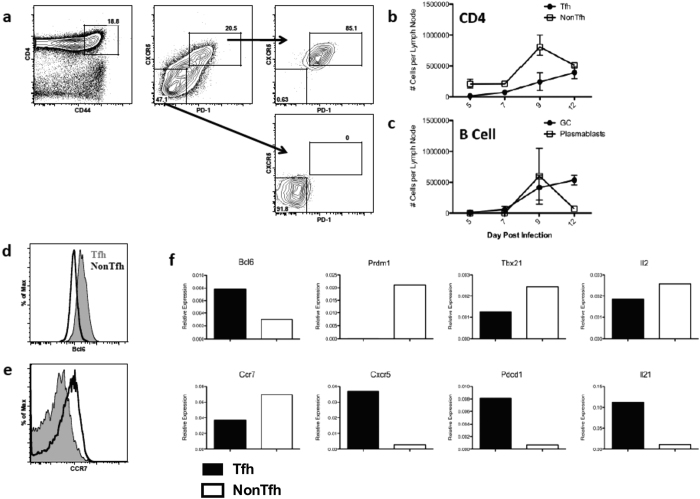
Tfh and NonTfh cells exhibit prototypical phenotype and kinetics following influenza infection. (**a**) Example of sorting strategy for isolating antigen-experienced Tfh and NonTfh cells after infection. SJL mice were infected intranasally with 50,000EID_50_ of A/New Caledonia/20/1999. Lymphocytes were isolated from the draining mediastinal lymph node on day 9 post infection and FACS sorted based upon expression of the following markers: CD4 + CD44hiCXCR5 + PD-1 + (Tfh) or CD4 + CD44hiCXCR5negPD-1neg (NonTfh). (**b,c**) Kinetics of effector CD4 T cell and B cell expansion following infection depicting Tfh and NonTfh populations, as well as GC (CD19 + B220 + GL-7 + CD95+) and plasmablasts (CD19 + B220intCD138+) (n = 2–5 mice per timepoint, representative of 2 experiments). (**d**) Expression of Bcl6 and (**e**) CCR7 in Tfh (Filled Grey) and NonTfh (Black Open) populations. (**f**) RT-PCR analysis of total RNA from Tfh (Filled) and NonTfh (Open) showing relative expression of various transcripts isolated from the indicated sorted T cell populations, normalized to B2m expression. Data shown is from a single experiment, representative of four.

We sought to evaluate partitioning of CD4 T cells into the Tfh vs NonTfh compartments during the primary immune response to intranasal infection of mice with influenza A virus. In order to survey any connection between specificity and function, we began by delineating the typical pattern of CD4 T cell expansion from naive to effector populations in a mouse model utilizing known specificities in the context of I-A^s^. This strain of mouse was chosen because of the broad peptide specificity that included more than 25 influenza-derived epitopes^25^. Mice were infected and the kinetics of CD4 T cell effectors, Tfh (CXCR5^+^ PD-1^+^) and NonTfh (CXCR5^−^PD-1^−^) (Fig. 1a), were monitored from day 5 to day 12 by flow cytometry (Fig. 1b). Prototypic markers of antigen-experienced T cells (CD44) and Tfh lineage commitment (CXCR5, PD-1) were used^1^,^2^,^6^,^26^,^27^,^28^. NonTfh populations expanded and exhibited a peak around day 9 and had begun to contract by day 12, while Tfh were still expanding at day 12 post infection (Fig. 1a,b). This accumulation of Tfh was accompanied by an increase in germinal center B cells from day 9 to 12 (Fig. 1c). From this, we chose day 9 to approximate a local maximum in both NonTfh and Tfh frequencies to best facilitate our ability to assay specificity and effector phenotype concurrently.

Further flow cytometric analysis was performed to determine levels of Bcl6 and CCR7 expression for both Tfh and NonTfh CD4 T cells. Tfh isolated from the mediastinal LN 9 days after infection exhibited heightened Bcl6 expression along with decreased CCR7 (Fig. 1d,e respectively) relative to the NonTfh population. Moreover, when the transcriptional profiles of Tfh and NonTfh were assessed by RT-PCR, Tfh expressed higher levels of mRNA encoding Bcl6, CXCR5, PD-1 (Pdcd1) and IL-21 and decreased levels of Blimp1 (Prdm1), Tbet and CCR7 compared to NonTfh (Fig. 1f), patterns that are consistent with these two distinct subsets of CD4 T cells. We then employed a flow cytometry-based sorting strategy where, on day 9 post infection, CD4 T cells were isolated from the draining LN of infected mice, enriched based on negative paramagnetic bead isolation and then sorted by flow cytometry based on expression of the cell-surface markers CD4, CD44 (to enrich for antigen experienced cells), CXCR5 and PD-1 (Fig. 1a). Sorted CD4 T cells were then assayed for peptide specificity in IL-2 ELISPOT assays by restimulation with individual influenza-derived peptides known to represent the major epitopes from this strain (A/New Caledonia/20/1999)^25^. Data in subsequent figures are reported as spots (IL-2) per million sorted cells (Tfh or NonTfh).

### NonTfh and Tfh populations exhibit epitope-specific differences in reactivity

**Figure 2:**
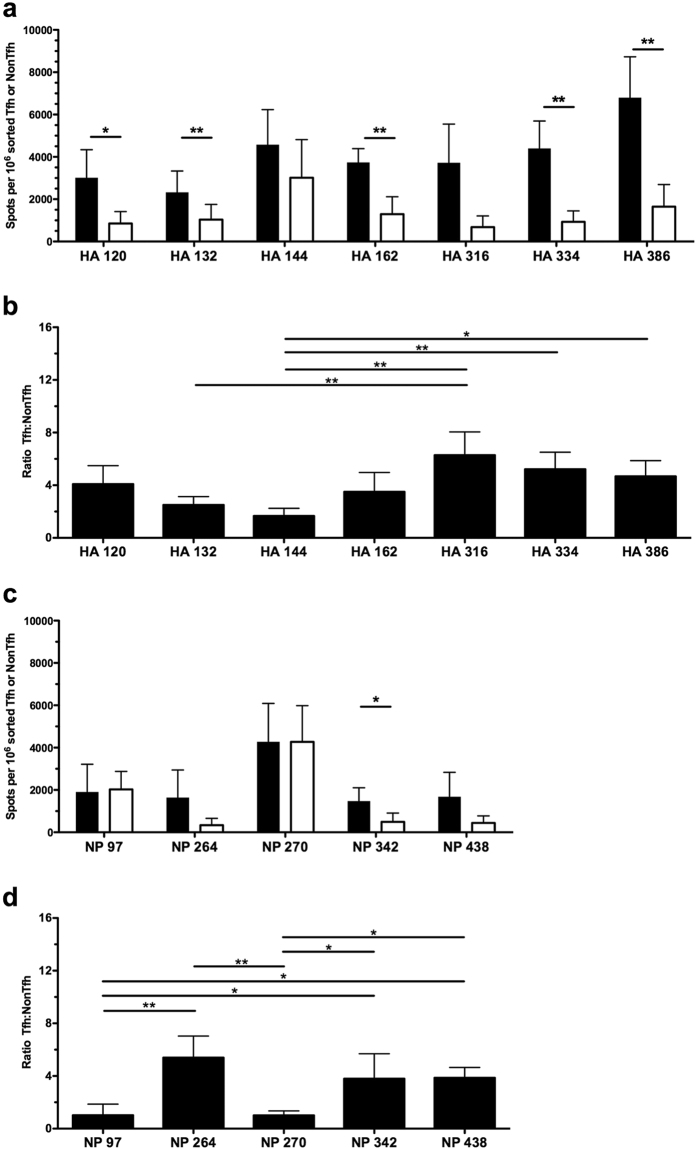
Influenza-specific effector cells distribute between Tfh and NonTfh depending on epitope specificity. Single epitope reactivity of sorted CD4 T cell populations at day 9 post infection, Tfh (Filled) and NonTfh (Open) from mediastinal lymph nodes pooled from 20–25 mice. CD4 T cell populations were assayed for epitope reactivity in IL-2 ELISPOT assays by restimulation with influenza-derived epitopes from HA (**a**) and NP (**c**). The epitope-specific IL-2 spots per million Tfh or NonTfh cells are shown. Relative distribution of antigen-reactive CD4 T cells between Tfh and NonTfh cells is depicted by the ratio of antigen-specific cells in the two populations for HA-specific T cells (**b**) and NP-specific cells (**d**). Mean and S.D. of 4 experiments. Statistical significance determined by two-tailed paired t test (**a**,**c**) and 1-way ANOVA with Tukey post test (**b**,**d**). *p < 0.05; **p < 0.01.

Hemagglutinin (HA) and nucleoprotein (NP) were chosen for this study because of the many readily detectable epitope specificities (5–7 per protein) and for the potential of new insight when studying two different classes of proteins. HA is a glycosylated, membrane surface protein with disulfide bonds, while nucleoprotein is an intracellular/internal protein in both the virion and infected cells. Any gross differences in Tfh reactivity to either protein may thus implicate antigen handling and acquisition as an important factor in the determination of T cell function. Sorted Tfh and NonTfh populations contained CD4 T cells reactive to all tested peptide epitopes in both HA and NP (Fig. 2a,c), demonstrating that neither NP- nor HA-specific T cells were excluded from Tfh or NonTfh CD4 populations.

Interestingly, although each epitope appeared capable of recruiting CD4 T cells into both the Tfh and NonTfh lineage, there were striking differences in the quantitative partitioning of epitope-specific T cells between Tfh and NonTfh populations. Of note, CD4 T cell distribution between Tfh and NonTfh was nearly equivalent for CD4 T cells specific for NP 97, NP 270 and HA 144 (Fig. 2a,c, respectively). In contrast, there were other peptide epitopes that recruited a much greater frequency into the Tfh population (HA 334, 386 and NP 264, 342 and 438) relative to the NonTfh populations.

To compare how a particular peptide-specific response partitioned into the Tfh or NonTfh populations, independently of the magnitude of each response, the frequency of antigen-reactive T cells in Tfh and NonTfh was calculated as a ratio for each epitope (Fig. 2b,d). This representation of HA- and NP-derived epitopes shows the two different patterns: some specificities are distributed selectively within the Tfh relative to the NonTfh population, while other populations of peptide-specific cells are virtually equal in distribution. Thus, these studies revealed an epitope-linked factor in the determination of T cell function. Since these variations in population-level distribution of antigen-specific T cells were observed in both proteins, it suggested that the peptide-epitope itself might be deterministic for CD4 T cell fate.

### Translation of antigen encounter from infection to protein vaccination retains epitope-specific patterns of specificity distribution

During the course of influenza infection, the abundance, kinetics of expression, availability, handling and processing of influenza antigen can vary among viral proteins. For example, membrane bound, glycosylated proteins such as HA and neuraminidase might be handled differently than soluble proteins such as NP and NS1 due to their potential to interact directly with antigen presenting cells by way of antigen targeting through pathways such as C-type lectin receptors^29^,^30^ some of which have been shown to specifically enhance Tfh responses^31^. Conversely, as a viral RNA binding protein, NP carries along its own innate activator that facilitates the stimulation of both TLR7 and RIG-I pathways^32^,^33^,^34^. Also, the infection itself elicits both a robust inflammatory response and the production of many innate mediators that might influence the developing immune response in the draining mediastinal lymph node. In order to diminish the contributions of many of these variables to the patterns of differentiation we had observed, we employed a vaccination regimen where equivalent amounts of recombinant protein (HA and NP) were co-delivered in emulsion of Incomplete Freunds Adjuvant (IFA) and lipopolysaccharide (LPS) into the footpad of mice. We expected this approach would normalize the kinetics, magnitude, costimulation and other protein-extrinsic factors that have the potential to influence the determination of CD4 T cell function. We included NP and HA together in the emulsion so that the diversity of antigen-specific cells is similar between infection and vaccination. NP and HA represent the major sources of immunodominant epitopes in this strain of mouse. The kinetics of the response to vaccination (Supplemental Figure 1) shows that at day 9, there are readily detectable Tfh and NonTfh populations. We also tested the distribution and abundance of Tfh and NonTfh over a 25-fold range of antigen (Supplemental Figure 2). These studies revealed that the abundance of each population of CD4 T cells increases with increasing immunizing dose but that the ratio of Tfh to NonTfh is preserved over this wide dose range. We chose to vaccinate with 5 μg of each protein to increase the yield of responding CD4 T cells, so that we could sample as many epitopes as possible.

**Figure 3:**
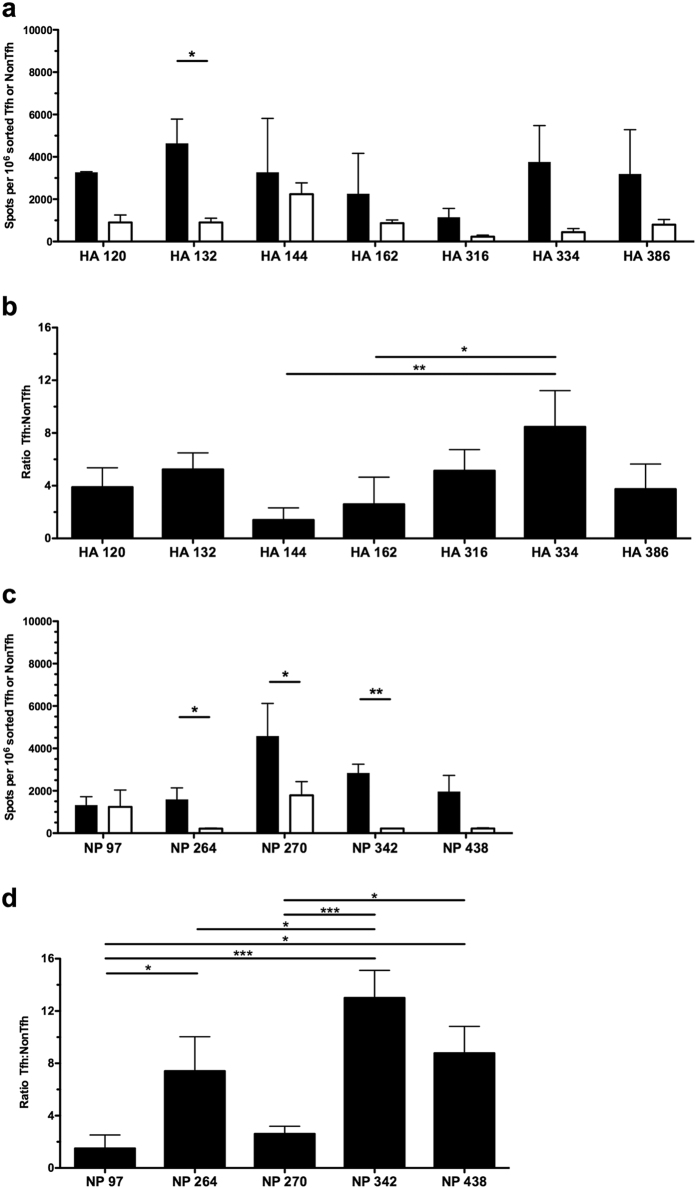
Vaccination with influenza proteins maintains epitope specific patterns in effector distribution between Tfh and NonTfh. Single epitope reactivity of sorted CD4 T cell populations at day 9 post vaccination, Tfh (Filled) and NonTfh (Open) from popliteal lymph nodes pooled from 10 mice. Mice were vaccinated subcutaneously with 5 μg HA and NP proteins together in IFA/LPS emulsion. CD4 T cell populations were assayed for epitope reactivity in IL-2 ELISPOT assays by restimulation with influenza-derived epitopes from HA (**a**) and NP (**c**). The epitope-specific IL-2 spots per million Tfh or NonTfh cells are shown. Relative distribution of antigen-reactive CD4 T cells between Tfh and NonTfh cells is depicted by the ratio of antigen-specific cells in the two populations for HA-specific T cells (**b**) and NP-specific cells (**d**). Mean and S.D. of 3 experiments. Statistical significance determined by two-tailed paired t test (**a,c**) and 1-way ANOVA with Tukey post test (**b**,**d**). *p < 0.05; **p < 0.01; ***p < 0.001.

As with infection, antigen-specific CD4 T cell reactivity to HA and NP epitopes was assayed from Tfh and NonTfh populations derived from the draining LN of vaccinated mice (Fig. 3a,c). As had been observed in response to influenza infection, antigen-reactive CD4 T cells partitioned between Tfh and NonTfh at ratios ranging from near equivalent (HA 144 and HA 162, NP 97) to those with a higher representation in the Tfh population (e.g. NP 264 and HA 334). The patterns detected with protein vaccination recapitulated many of those observed in the context of infection, suggesting that the milieu associated with infection was not a deciding factor in determining the distribution of CD4 T cells between Tfh and NonTfh populations. Further underscoring the preservation of epitope-specific differences in T cell distribution, representation of the antigen reactive Tfh and NonTfh populations as a ratio (Fig. 3b,d) indicates that several epitopes are much better suited for driving CD4 T cells towards a Tfh function (HA 334, NP264, NP438) or toward a NonTfh function (HA 144, NP 97).

### Effector function is determined by the epitope specificity and is largely independent of protein context

Though we made efforts to normalize the way in which HA and NP protein antigen were acquired and processed by antigen presenting cells, properties intrinsic to each protein could still be a determining factor in the generation of CD4 T cell effector function that could influence epitope dependent patterns of differentiation. For example, B cell receptor binding has been shown to both suppress or enhance the release and presentation of peptides from native protein^35^,^36^, which could influence the relative levels of surface peptide:MHC complexes for any given epitope on B cells. These antigen-processing events might influence quantitative aspects of B cell epitope display and thus stable commitment of a CD4 population to the Tfh lineage. Also, protein structure and competing peptides might also influence the epitope-specific patterns in the CD4 T cell response.

**Figure 4:**
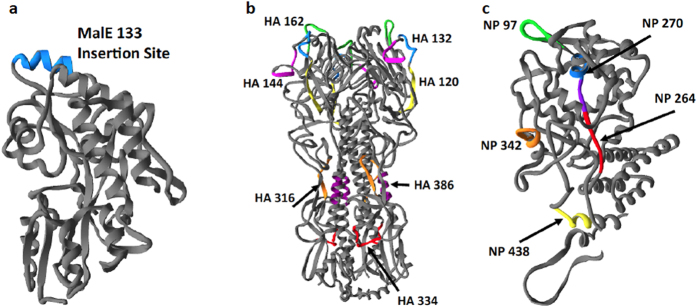
Epitope locations within the native protein context of hemagglutinin and nucleoprotein and the heterologous protein MalE133. Protein structures of MalE133 (**a**), hemagglutinin (**b**) and nucleoprotein (**c**). Influenza epitopes and their locations are indicated by coloration and are denoted by first amino acid associated with the 17-mer peptide used for testing of CD4 T cell reactivity. For MalE133 protein, a heterologous peptide is shown for illustrative purposes at the insertion site within the protein structure. The structures used to model peptide epitope location were sufficiently homologous to H1N1 A/New Caledonia/20/1999 proteins to overlay New Caledonia epitopes: Hemagglutinin (HA0) from A/South Carolina/1/18 (H1N1); Nucleoprotein from A/England/256/2009 (H1N1).

To control for any differences dictated by antigen structure and immunogenicity, peptides that represented the most striking disparities in Tfh differentiation from HA and NP were selected and engineered into the exact same insertion site within a heterologous protein context. The maltose binding protein (MalE) of *E. coli* was chosen for this because it has been shown to accommodate foreign peptide inserts without compromising overall protein structure^37^,^38^. DNA-encoded peptide sequences of ~17 residues were inserted into a MalE expression vector, which was in turn transformed into *E. coli* for protein production. With each influenza-derived epitope positioned within and flanked by the same protein structure, differences in antigen handling by dendritic cells or B cells due to native protein properties would be minimized. Figure 4a displays the site used for insertion of the peptides into MalE at amino acid 133. For comparison, the localization of each epitope within HA (Fig. 4b) and NP (Fig. 4c) is also denoted to accentuate the wide distribution of epitopes throughout the native proteins and within various types of secondary structure: helices, sheets and non-structured loops.

**Figure 5:**
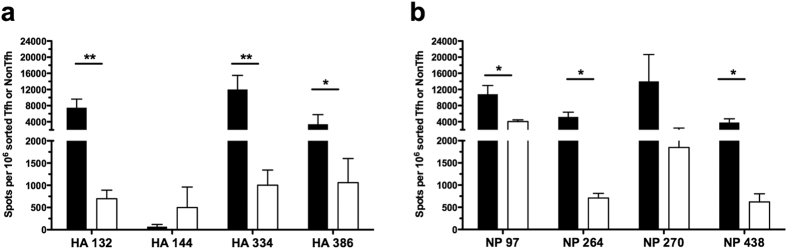
Epitope-specific reactivity for Tfh and NonTfh following vaccination with a heterologous protein containing influenza-derived epitopes. Shown are the responses of sorted Tfh and NonTfh cells to HA-derived epitopes (**a**) and NP-derived epitopes (**b**). As before, mice were vaccinated with the indicated individual MalE133 constructs (5 μg protein) in an IFA/LPS emulsion. Cells were isolated from popliteal lymph nodes pooled from 5 mice per protein at day 9 post vaccination. Eight different MalE133 proteins containing influenza epitopes were constructed. Mean and S.D. of 3–5 experiments. Statistical significance determined by one-tailed paired t test. *p < 0.05; **p < 0.01.

**Figure 6:**
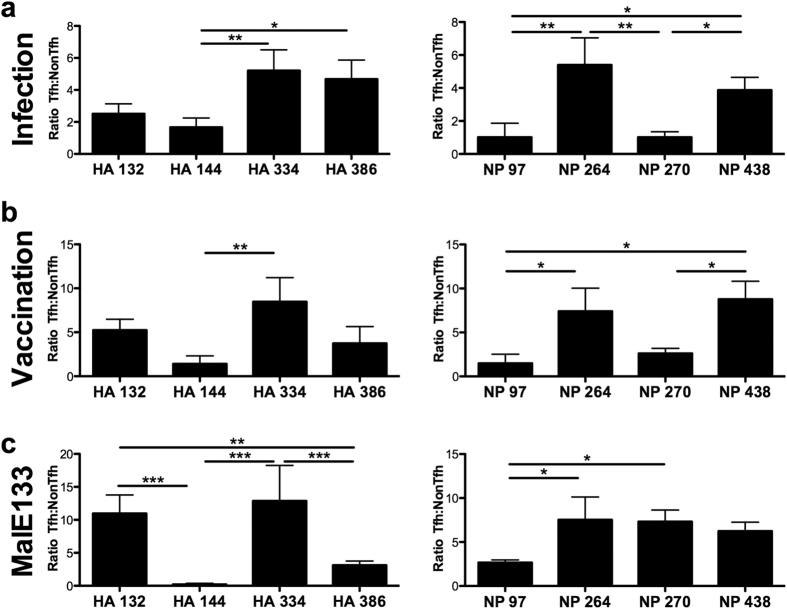
Context-independent relationship between epitope-specificity and effector function. Epitope-specific reactivity for individual specificities were compared as ratios of reactive Tfh:NonTfh as shown previously for (**a**) infection (Fig. 2) and (**b**) HA/NP vaccination (Fig. 3). Data from previous figures on infection and vaccination were duplicated below for comparison of the response to the inserted epitope in MalE. (**c**) The ratio of reactive cells (Tfh:NonTfh) for each epitope is depicted for MalE133 vaccination, as calculated from enumeration of IL-2 producing cells in each population (Fig. 5). Statistical significance determined by 1-way ANOVA with Tukey post test. *p < 0.05; **p < 0.01; ***p < 0.001.

Mice were vaccinated with the recombinant MalE constructs containing the inserted influenza peptide epitope in an IFA/LPS emulsion. Post vaccination, sorted Tfh and NonTfh populations from the draining lymph node were assayed for epitope-specific reactivity (Fig. 5a,b) using cytokine ELISPOTs. These experiments revealed that vaccination with the MalE construct was sufficient to drive both Tfh and NonTfh differentiation for each epitope-specific T cell population. Importantly, many of the patterns observed for different influenza derived epitopes were largely preserved in this new context. To illustrate the degree to which individual epitopes differed in the ability to generate either Tfh or NonTfh, the spot counts were again converted to a ratio, as before, of antigen specific Tfh:NonTfh (Fig. 6c). To gain insight into how the epitope-specific CD4 T cells partitioned into Tfh and NonTfh within each of the different priming conditions, data for the corresponding epitopes in the context of infection (Fig. 6a) or HA/NP vaccination (Fig. 6b) were extracted and represented alongside data from the MalE constructs for comparison. Strikingly, for many epitopes, there was notable preservation of the correlation of antigen-specificity to effector function first observed with infection. When considered as a rank order within each of the approaches used, HA 334, NP 264, NP 438 elicit a dominant Tfh pattern while HA 144 and NP 97 consistently display the least ability to promote a Tfh response that was also demonstrated with purified protein, and finally with the epitope imported to a completely different antigenic context. There were epitopes whose responses were altered, especially when in MalE. In particular two epitopes (HA 386 and NP 270) progressively increased their tendency to drive a Tfh response from infection, to protein vaccination to MalE vaccination. We speculated HA 132 and HA 270 peptides drew a more focused high affinity TcR response when they were encoded within MalE relative to infection. To evaluate this, we compared the “functional avidity” of CD4 T cells specific for these epitopes when elicited by infection vs. when encoded in MalE. When in MalE, these epitopes elicited a response within the range detected in infection but at the higher end of the range (Supplementary Fig. S3). Thus, it is possible that the MalE constructs of these peptides recruit a subset of CD4 T cells of somewhat higher affinity than those generated by infection. However, it is also possible that, although the individual MalE constructs have largely the same structure, the inserted epitopes may have altered B cell recognition. Collectively, the results using the three different contexts of priming suggest that in addition to the microenvironment in which CD4 T cell priming occurs, the specificity of a polyclonal CD4 T cell population can have a profound influence on the ultimate effector fate of the CD4 T cell population.

### The impact of functional avidity in partitioning of CD4 T cells in the polyclonal responses between Tfh and NonTfh cells

**Figure 7:**
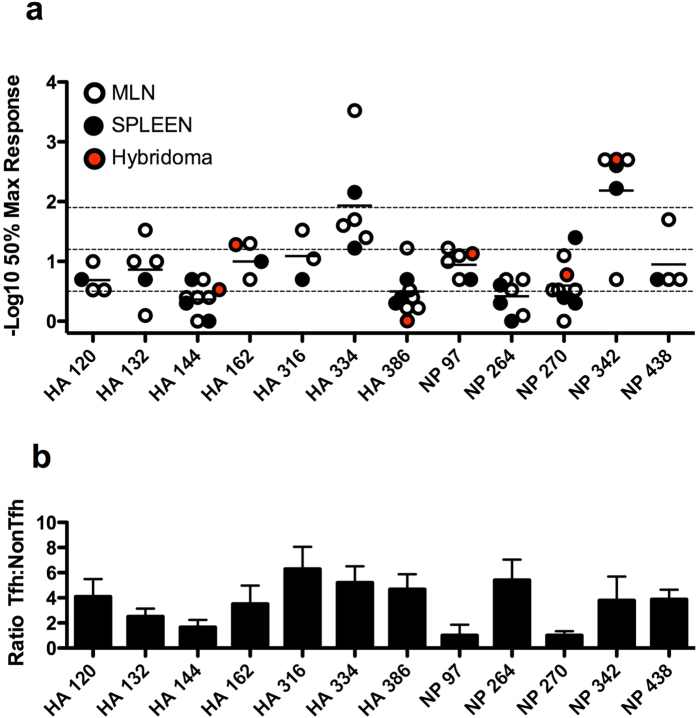
The impact of functional avidity in partitioning of polyclonal CD4 T cells between Tfh and NonTfh. Cells isolated from either MLN (open symbol) or spleen (closed symbol) at day 9 or 10 post-infection were evaluated by ELISPOT assay for reactivity to the indicated influenza peptide epitopes. The affinity of the polyclonal CD4 T cell response to various influenza epitopes was determined as the peptide dose at which 50% maximum reactivity was reached, and is indicated here as the negative log10 of this value (**a**). Mean of MLN and spleen derived values in independent assays (n = 3–8) is indicated. For reference, the dotted line at 0.5 is associated with a 0.32 μM peptide dose, 1.2 with 0.063 μM and 1.9 with 0.013 μM, roughly approximating low, medium and high affinity, respectively. Hybridomas derived from CD4 T cells isolated from infected mice were also evaluated to approximate TCR affinity, measured as the peptide concentration at which reactivity was first detectable in an ELISPOT assay. Mean values (red symbol) were determined from 3 (HA 162, 386, NP 342), 7 (NP 97), or greater than 10 (HA 144, NP 270) individual clones. Data from panels Fig. 2b,c have been combined and reproduced here (**b**) for comparison between Tfh/NonTfh ratio and CD4 T cell affinity.

Several recent studies have pointed to high TcR affinity and/or optimal dwell time between the TcR and MHC as a factor that controls commitment to Tfh lineage^22^,^23^. We sought to address this issue by assessing the “functional avidity” of the CD4 T cells specific for the large and diverse set of peptide epitopes identified after infection that showed distinct partitioning between Tfh and NonTfh (Fig. 2). Mice were infected and purified CD4 T cells were scored for their relative affinity by performing peptide dose response assays, coupled with cytokine ELISPOTs. Figure 7a shows the results of independent assays for each epitope, as well as the results from CD4 T cell hybridomas that were available for some of the epitopes. Shown for comparison in Fig. 7b is the ratio of the Tfh to NonTfh elicited by these peptides after infection. These data revealed that there was a range of affinities of the CD4 T cells specific for different peptides. We have operationally divided them into low (0.5), medium (1.2) or high (1.9), which corresponds to 50% response achieved at approximately 0.32 μM, 0.063 μM or 0.013 μM, respectively. Among those with the highest apparent affinity were CD4 T cells specific for HA 334 and NP 342, both favored to become Tfh. In contrast, the CD4 T cells specific for HA144 have the lowest apparent affinity and these cells are poorly represented as Tfh. However, there are epitopes that do not fit this relationship between avidity and the likelihood of the population of epitope-specific CD4 T cells to become Tfh. For example, CD4 T cells specific for HA 162, HA 316 and NP 97 have quite similar intermediate apparent functional affinities but only HA 162 and HA 316 are enriched for Tfh, while NP 97 specific cells display the lowest tendency to differentiate into Tfh. Similarly, the population of CD4 T cells specific for NP 264 and HA 386 have very low apparent functional avidity and yet they drive a strong Tfh response. We conclude that multiple factors dictated by the pMHC complex are likely responsible for the epitope-dependent patterns in partitioning of cells within the Tfh or the NonTfh compartments during an immune response.

## Discussion

In this study, we have demonstrated a link between CD4 T cell specificity and function that transcends extrinsic influences such as cytokine and costimulatory signaling. Examination of the distribution of antigen-specific CD4 T cells between Tfh and NonTfh populations after infection revealed the potential relationship between specificity and function, with some epitope-specific populations favoring Tfh function over NonTfh. When examined outside of the context of infection, the specificity/function relationship was largely maintained after vaccination with recombinant protein. Furthermore, the potential effects of antigen handling and processing that might differentially influence epitope presentation were bypassed by testing each epitope in the same antigenic context through the importation of the peptide sequence into the heterologous protein MalE, which offers a distinct set of competing peptides and three dimensional structure.

Early signaling events have been shown to play a critical role in the “decision” to become a Tfh, with the fate being set as early as two cell divisions after activation^11^,^39^. Tfh and NonTfh effector function is determined by the differential regulation of several important factors not limited to CXCR5, CCR7, Bcl6, Blimp1, CD25 and ICOS. What is less well understood is the role that signaling through the TCR has upon downstream events that lead to the choice between a Tfh and NonTfh fate for CD4 T cells leading to Tfh differentiation, with research directly addressing this question being quite limited. In one study involving the CD4 T cell response to pigeon cytochrome *c* in TcR transgenic mice, it was shown that TcRs with higher affinity with peptide:MHC were more likely to generate a Tfh phenotype^23^. In a second recent study by Jenkins and coworkers^22^, the specificity of clonal CD4 T cells was shown to be a determining factor in functional outcome, related to the quality of interaction between the TCR and peptide:MHC complex, described as the “dwell time” that the tri-molecular complex was engaged. Their data was most consistent with the scenario in which the degree of clonality of a responding population can impact the functional outcome. As such, CD4 T cells from a given clone could display propensities to differentiate toward a given effector outcome, but as the number of distinct precursor CD4 T cell clones was increased, the phenotype tended to average the preferences detectable within single clones. Here we have shown that within multiple proteins and environmental contexts, with foreign peptides that should recruit a broad repertoire of CD4 T cells, the Tfh/NonTfh distribution among the responders drawn from the endogenous TcR repertoire depended on peptide specificity.

We have considered the possibility that the responding TcR repertoire restricted by I-A^s^ may be small relative to alternative alleles of class II expressed in other strains of mice. Amino acid substitutions within the peptide-binding groove of I-A^s^, particularly near the C-terminal anchor position, may influence epitope selection to such a degree that the overall CD4 T cell repertoire is limited. The substitutions found in this particular allele of MHC-II (αN69T, βW61Y) are associated with a disruption in hydrogen bond formation between peptide and the MHC binding groove^40^ that might limit the potential for peptide binding, and favor a very specific set of amino acids that could drive either TcR repertoire selection or peripheral T cell priming^41^. We have not been able to determine the degree of diversity that compromises the responder population for the epitopes we have studied here due to a lack of tetramers for the allele of MHC class II molecules (I-A^s^) expressed by this mouse or other means to purify peptide specific cells prior to TcR sequencing. It will clearly be of value to extend these studies to class II molecules that elicit a broad repertoire of CD4 T cells and that are amenable to peptide exchange and stable tetramer derivation.

We evaluated the contribution of TcR affinity to the different patterns of differentiation observed among the different epitopes. These studies revealed that some of the epitope-specific patterns were consistent with the paradigm that affinity of peptide:MHC for the responding TcR repertoire is an important factor in determining Tfh lineage commitment. However, within the limits of the assay performed, there were a number of epitopes for which apparent affinity was not a good predictor of Tfh lineage commitment. Based on these experiments, we conclude that while affinity of the TcR for peptide:MHC may be one, perhaps the first, permissive step in the option to become a Tfh, there is likely at least one other checkpoint that can enforce or diminish continuance along this pathway of differentiation and that may be playing a role in the distinctive patterns we have observed among the epitopes studied. It is clear that epitope display by B cells under physiological conditions is needed for the CD4 T cells to commit to the Tfh lineage. This might easily be envisioned to differ among the peptides studied. Ig receptor binding to antigen can decrease or increase B cell epitope display^35^, likely by altering accessibility or processing of the internalized antigen into peptides. B cell antigen presentation will also be modulated by the expression of DO, an inhibitor of DM editing^42^,^43^,^44^,^45^. The impact of DO is thus likely to influence the epitope abundance on antigen-specific B cells. We speculate that together, these check points contribute to the distinctive patterns observed among the epitopes studied. Because Tfh continue to interact with antigen-bearing B cells throughout the germinal center response, it would be of interest to determine whether the epitope specificity of Tfh changes over time, or with purification of “germinal center” Tfh. The specificity may change, as antigen becomes progressively limiting and as DO expression diminishes in germinal center B cells^46^. It would also be of value to understand the role of inter-epitope competition on Tfh selection. The MalE vector has a similar diversity and abundance of CD4 T cells so that the influence of these factors was not evaluated here.

Overall, these studies suggest that prediction of the response phenotype of the endogenous CD4 T cell repertoire will depend on understanding the microenviroment in which the response is initiated, the diversity of the epitopes recognized by the CD4 T cells and how they are selected at the initial priming step and in subsequent requisite interactions with antigen presenting cells. The studies reported here may also have an impact on vaccine strategies. Could the frequency of a particular CD4 T cell specificity serve as a predictor of later B cell responses, or perhaps a response more dedicated to protection from infection in distal tissues? The ability of peptide-specific T cells to sustain their characteristic commitment toward Tfh or NonTfh function through different types of vaccinations and infections that generate distinct priming microenvironments could be exploited for directed vaccination with the goal of a targeted effector response.

## Materials and Methods

### Mice

Female SJL (I-A^s^) mice were obtained from the National Cancer Institute (Frederick, MD) and maintained in a specific-pathogen-free facility at the University of Rochester Medical Center, according to institutional guidelines. All animal protocols used in this study adhere to the AAALAC, International, the Animal Welfare Act and the PHS Guide, and were approved by the University of Rochester Committee on Animal Resources, Animal Welfare Assurance Number A3291-01. The protocols under which these studies were conducted were originally approved on 4 March 2006 (protocol no. 2006–030) and have been reapproved every 36 months with the most recent review approval occuring on 6 February 2015. Mice in all groups were age matched, and used at 6 to 12 weeks of age.

### Infections and Vaccinations

Mice were infected with A/New Caledonia/20/1999 virus prepared from the allantoic cavity of embryonated chicken eggs (described previously^47^) at a dose of 50,000EID_50_, inoculated intranasally in phosphate buffered saline (PBS). For vaccination studies, mice were administered an emulsion of Incomplete Freunds Adjuvant (IFA, Sigma), lipopolysaccharide (0.6 μg/mL) and protein in PBS. Mice received 5 μg each of recombinant hemagglutinin, H1 A/New Caledonia/20/1999 (BEI Resources) and 5 μg recombinant nucleoprotein (prepared in house as described previously^48^). In experiments involving MalE133 protein, mice received 5 μg of protein. Emulsion was administered subcutaneously in each rear footpad in a volume of 50 μL per foot. MalE133 proteins containing the peptide epitopes corresponding to HA 132, HA 144, HA 334, HA 386, NP 97, NP 264, NP 270 and NP 438 were incorporated individually within the MalE133 protein (position 133) as previously described^38^.

### Cell Isolation and Staining for Flow Cytometry

Mice were euthanized at the indicated times post infection or vaccination and CD4 T cells were isolated from the draining lymph node (mediastinal or popliteal). Tissues were prepared as a single cell suspension and depleted of RBC by treatment with ACK lysis buffer (0.15 M NH_4_Cl, 1.0 mM KHCO_3_, 0.1 mM Na_2_-EDTA in H_2_O, pH 7.2) when necessary. Cell suspensions were then enriched for CD4 T cells by use of Magnetic-Activated Cell Sorting (MACS) (CD4 T Cell Isolation Kit II, Miltenyi Biotec, Auburn, CA). Cells were then stained for FACS sorting, first in Fc Block (BD Biosciences) followed by addition of anti-CD4 (RM4-5), anti-CD44 (IM7), anti-PD-1 (J43, eBiosciences) and biotinylated anti-CXCR5 (2G8). All antibodies were purchased from BD Biosciences except where noted. Secondary staining with PE-Streptavidin (BD Biosciences) was necessary to detect the anti-CXCR5 antibody. Stained cells were sorted using a FACSAria (BD Biosciences) to isolate CD4 + CD44hiCXCR5 + PD-1 + Tfh cells or CD4 + CD44hiCXCR5negPD-1neg NonTfh cells. Analytical staining on lymph node cells for various B cell and CD4 T cell populations was performed using antibodies specific for the aforementioned markers, as well as: Bcl6 (K112-91), CCR7 (4B12), B220 (RA3-6B2), CD19 (1D3), CD138 (281-2), GL7 and CD95 (Jo2). Live cells populations were determined by staining with Live/Dead fixable viability dye (Life Technologies), or 7AAD (BD Biosciences). For intracellular staining of the transcription factor Bcl6, the Transcription Factor Buffer Set (BD Biosciences) was used according to the manufacturers instructions. Analytical samples were run on a FACSCanto II system using FACS Diva software (BD Biosciences), and analyzed using FlowJo software, version 8.8.6 (Tree Star, Inc.).

### ELISPOT assays

CD4 T cell peptide specificity was determined via IL-2 ELISPOT assay as described previously^49^. Briefly, sorted CD4 + CD44hi Tfh or NonTfh cells were plated with syngeneic splenocytes and restimulated with influenza-derived peptides (see below). T cells were plated at a density appropriate for eliciting spots that would be in a readable range (50–250 spots). For measurement of apparent affinity, CD4 T cells were assayed for specificity using peptide-dose response ELISPOT assays. Cells were evaluated for reactivity across a 6-log range of peptide beginning at 10 μM, with the peptide concentration corresponding to 50% maximum reactivity being used as an approximation of affinity. Spots were enumerated on an Immunospot Reader Series 2A using Immunospot software (v5.0.9.19) and converted to IL-2 spots per million T cells with background (T cells, APC and no restimulation) subtracted. Data is also presented as a ratio of reactive cells in the Tfh compared to NonTfh populations.

### Synthetic peptides

Peptides used for ELISPOT assays were derived from a set of 17-mer peptides that encompassed the entire sequence of hemagglutinin (HA) from influenza A/New Caledonia/20/1999 virus (H1N1) and nucleoprotein (NP) from influenza A/New York/348/2003 virus (H1N1), which is highly conserved compared to A/New Caledonia/20/1999. Peptide arrays were obtained from the NIH Biodefense and Emerging Infections Research Repository (NIAID) as follows: NR-2602 for the HA protein of A/New Caledonia/20/1999 and NR-2611 for the NP protein of A/New York/348/2003. Single peptides were used at a final concentration of 10 μM. Peptide epitopes, delineated previously by our lab where the peptide number refers to the first amino acid in the published sequence^25^, and as provided by the repository, are as follows: HA 120 [EQLSSVSSFERFEIFPK], HA 132 [EIFPKESSWPNHTVTGV], HA 144 [TVTGVSASCSHNGKSSF], HA 162 [RNLLWLTGKNGLYPNLS], HA 316 [IGECPKYVRSAKLRMVT], HA 334 [LRNIPSIQSRGLFGAIA], HA 386 [NAINGITNKVNSVIEKM], NP 97 [YKRVDGKWVRELVLYDK], NP 264 [LILRGSVAHKSCLPACV], NP 270 [VAHKSCLPACVYGPAVA], NP 342 [RVSSFIRGTRVLPRGKL] and NP 438 [SDMRAEIIKMMESARPE].

### Real-Time PCR

Total RNA, extracted with TRIzol (Life Technologies, Carlsbad, CA) and prepared according to the manufacturer’s instructions, was purified from sorted cells and used to synthesize cDNA. TaqMan Gene Expression Master Mix and the following TaqMan Gene Expression assays were obtained from Life Technologies: Bcl6 (Mm00477633_m1), B2m (Mm00437762_m1), Ccr7 (Mm01301785_m1), Cxcr5 (Mm00432086_m1), Il2 (Mm00434256_m1), Il21 (Mm00517640_m1), Pdcd1 (Mm00435532_m1), Prdm1 (Mm01187285_m1), Tbx21 (Mm00450960_m1). Real-time PCR reactions were run in triplicate on the QuantStudio 12KFlex System (Life Technologies). Data were analyzed with ExpressionSuite Software v1.0.3 (Life Technologies), and normalized to B2m expression. Synthesis of cDNA and analysis for quality, as well as the RT-PCR plate run were performed by the University of Rochester Genomics Research Center.

### Protein Modeling

The structures of hemagglutinin (1RD8)^50^, nucleoprotein (4DYS)^51^ and MalE133 (1IUD)^37^ were all analyzed for the internal positioning of CD4 T cell epitopes. Protein structures were acquired from the Protein Data Base (PDB ID are indicated) and modified in Swiss-Pdbviewer.

### Statistical Analysis

All statistical analyses were performed using GraphPad Prism software version 5.0a, (GraphPad Software, Inc, La Jolla, CA). Statistical test used is indicated for each data set, with significance determined as p value < 0.05.

## Additional Information

**How to cite this article**: Knowlden, Z. A. G. and Sant, A. J. CD4 T cell epitope specificity determines follicular versus non-follicular helper differentiation in the polyclonal response to influenza infection or vaccination. *Sci. Rep.* **6**, 28287; doi: 10.1038/srep28287 (2016).

## Supplementary Material

Supplementary Information
