# Supplementary material for: CD4 T cell epitope specificity determines follicular versus non-follicular helper differentiation in the polyclonal response to influenza infection or vaccination
Source: Sci Rep. 2016 Jun 22;6:28287. doi: 10.1038/srep28287 (PMC4916409; doi:10.1038/srep28287)
Supplement: Supplementary Information [file srep28287-s1.doc]

**Supplementary Information**

**CD4 T cell epitope specificity determines follicular versus non-follicular helper differentiation in the polyclonal response to influenza infection or vaccination**

Authors: Zackery A. G. Knowlden; Andrea J. Sant

**SUPPLEMENTARY INFORMATION**
**
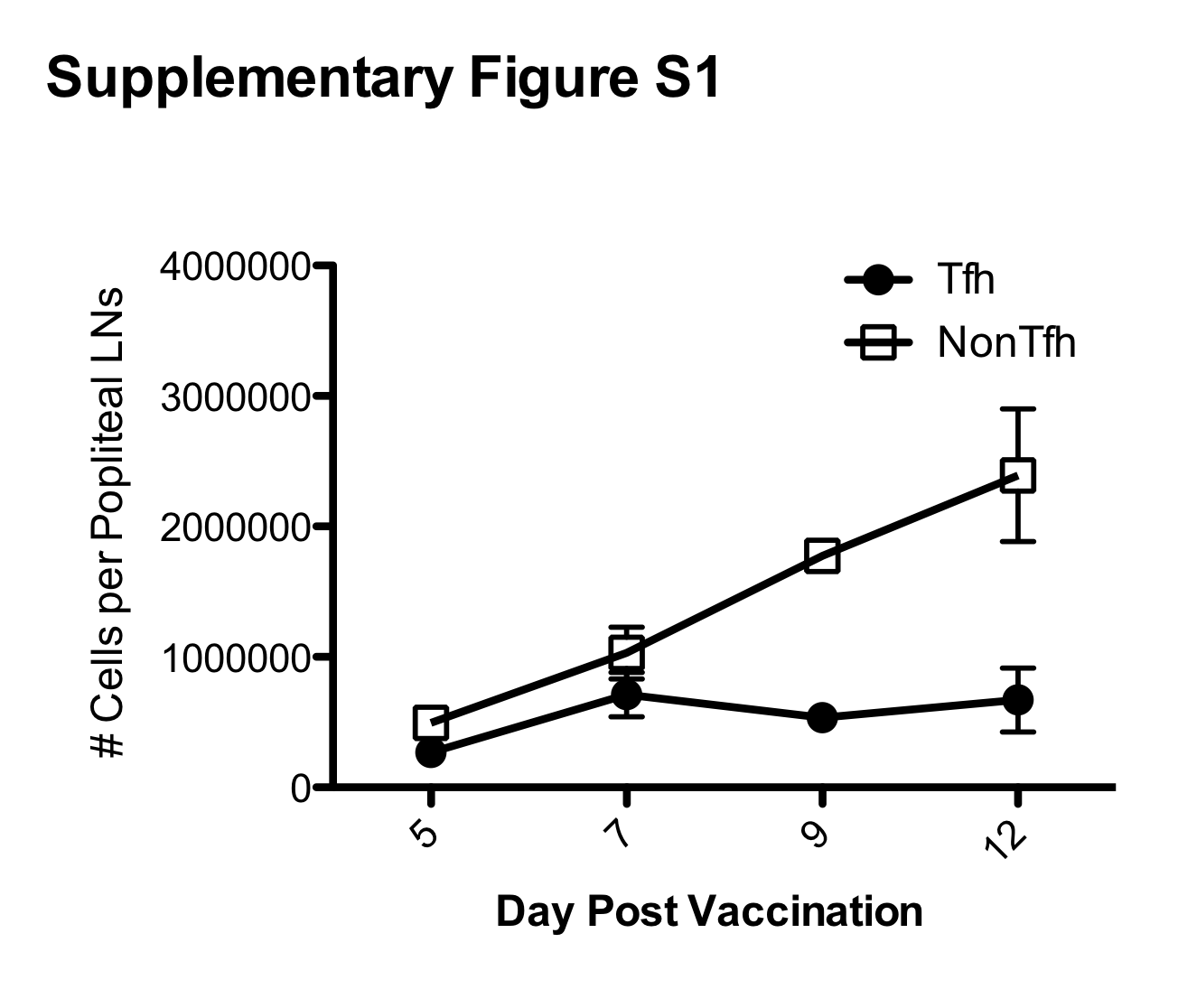
**

**Supplementary Figure S1. Kinetics of Tfh and NonTfh expansion following protein vaccination.** The number of Tfh (Filled) and NonTfh (Open) cells in the draining popliteal LN was determined after subcutaneous footpad vaccination with HA and NP protein (5μg each) in an emulsion of IFA and LPS. These populations were tracked via flow cytometry at the indicated days following vaccination. Shown are the numbers of Tfh or NonTfh in the two pooled PLN, mean and S.D. of three individual mice per day.


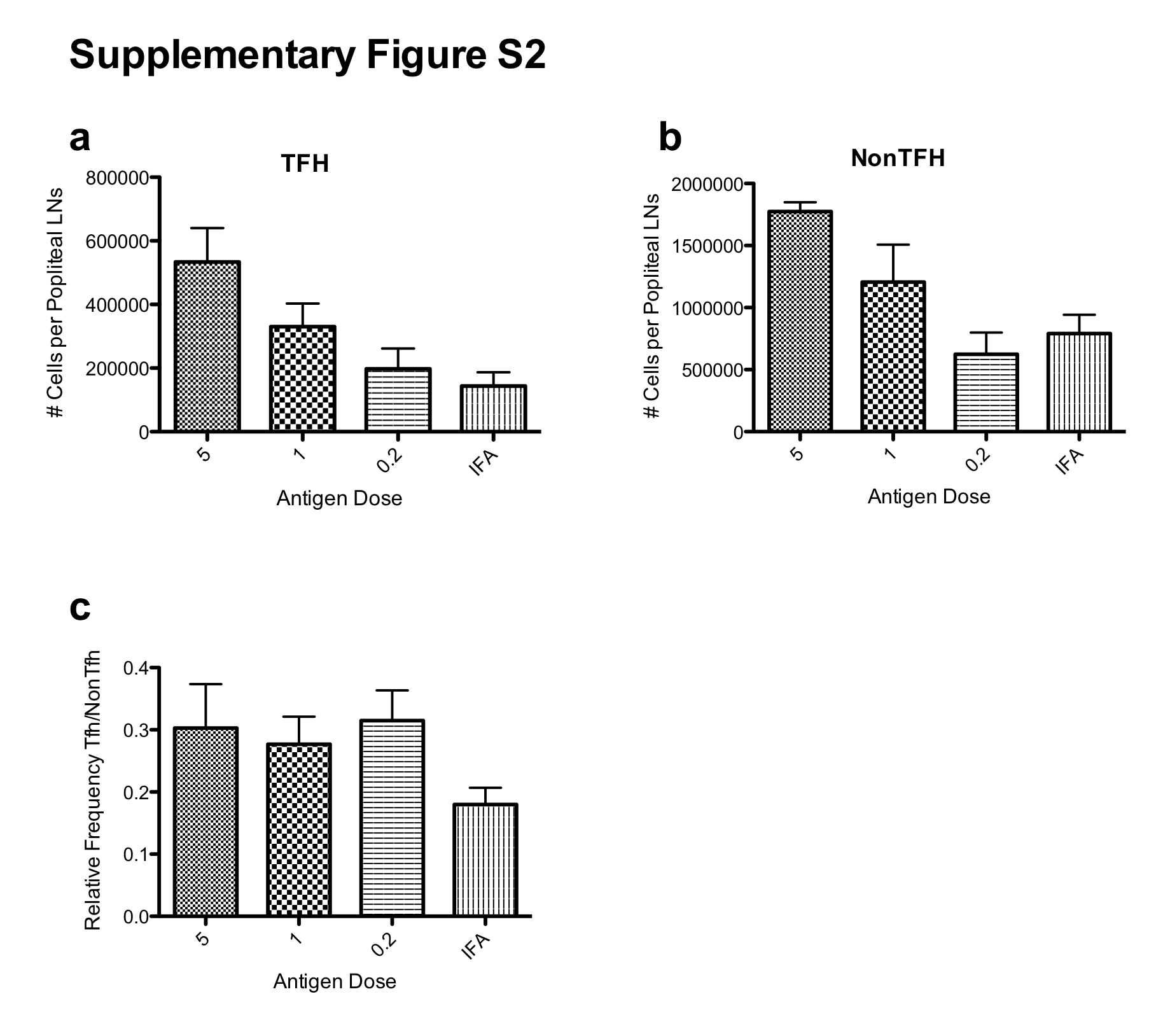


**Supplementary Figure S2. Protein antigen dose influences the scale of the CD4 effector T cell response.** Frequency of Tfh and NonTfh within the popliteal LN of vaccinated mice was determined at day 9 post vaccination. Mice received the indicated dose of protein (5/1/0.2μg each of HA and NP) in emulsion of IFA and LPS. Pooled PLN were enumerated by flow cytometry for Tfh (**a**) or NonTfh (**b**). Also indicated is the number of effector cells from mice that received IFA/LPS emulsion with no protein (IFA). The number of Tfh and NonTfh were compared as a ratio (**c**) to determine the relative frequency of these two populations across all antigen doses. Shown are the numbers of Tfh or NonTfh in the two pooled PLN, or relative frequency, mean and S.D. of three individual mice per antigen dose.

**
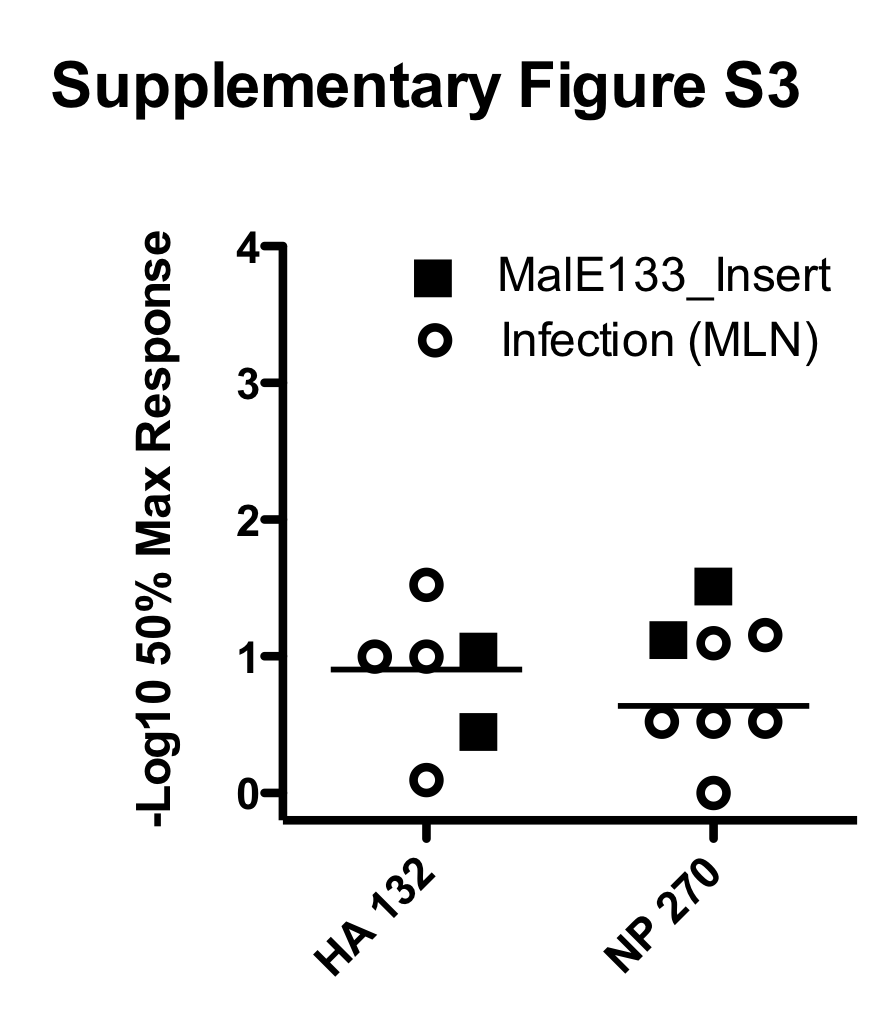
**

**Supplementary Figure S3. Affinity of the polyclonal CD4 T cell response to HA 132 and NP 270 following MalE133 vaccination.** The approximate affinity of CD4 T cells reactive to either HA 132 or NP 270 was determined as the peptide concentration at which 50% maximum reactivity was achieved. CD4 T cells derived from the PLN of MalE133_HA132 or MalE133_NP270 vaccinated mice (n = 2-3) were evaluated. Shown is the negative log 10 value from two experiments (filled squares) overlaid on the data derived from Figure 7 (MLN only) for these two epitopes.
